# Supplementary material for: Evaluation of the Diagnostic Accuracy of Nasal Cavity and Nasopharyngeal Swab Specimens for SARS-CoV-2 Detection via Rapid Antigen Test According to Specimen Collection Timing and Viral Load
Source: Diagnostics (Basel). 2022 Mar 14;12(3):710. doi: 10.3390/diagnostics12030710 (PMC8947492; doi:10.3390/diagnostics12030710)
Supplement: Supplementary file 1 [file diagnostics-12-00710-s001.zip › diagnostics-1637220-supplementary.pdf]

Table S1. Comparison of Ct values of E gene and RdRp gene in positives and negatives read of rapid antigen test (RAT) from two different location of sample collection.

	Nasopharyngeal Specimen			Nasal Cavity Specimen		
**Ct value**	**Negatives**	**Positives**	***p***	**Negatives**	**Positives**	***p***
***E* gene**						
Mean (SD)	29.2 (4.0)	18.5 (5.2)	<0.01	27.5 (5.3)	18.4 (5.3)	<0.01
***RdRp* gene**						
Mean (SD)	27.9 (4.0)	17.1 (5.2)	<0.01	26.3 (5.2)	16.9 (5.3)	<0.01
